# Fantastic wetlands and why to monitor them: Demonstrating the social and financial benefit potential of methane abatement through salt marsh restoration

**DOI:** 10.1371/journal.pclm.0000317

**Published:** 2024-07-05

**Authors:** Adam V. Reilly, Nathaniel H. Merrill, Kate K. Mulvaney, Phil Colarusso, Erin Burman

**Affiliations:** 1 Region 1, U.S. Environmental Protection Agency, Boston, Massachusetts, United States of America,; 2 Office of Research and Development Atlantic Ecology Division, U.S. Environmental Protection Agency, Narragansett, Rhode Island, United States of America,; 3 Oak Ridge Institute of Science and Education (ORISE) Research Fellow, Boston, Massachusetts, United States of America

## Abstract

Salt marsh restoration has the potential to reduce greenhouse gas emissions thereby providing an opportunity for blue carbon crediting, but implementation has been limited to date because of insufficient data and validation. In this paper, we demonstrate the potential scale of methane emissions that could be avoided if salinity-reducing impairments are mitigated by applying findings from six salt marsh restoration sites in Massachusetts combined with a previously demonstrated application of the salt marsh salinity-methane relationship. We used calculations of these avoided emissions to estimate the social benefit of salt marsh restoration by calculating avoided costs. Of the six sites selected, restorations at two sites were successful in improving salinity which we used as a methane proxy. Our approach and findings demonstrate the potential benefits in developing consistent accounting methodologies to better track, prioritize, and implement wetlands restoration strategies to mitigate methane emissions and contribute toward state-level emissions reduction targets across some of the 475 Massachusetts salt marches with an existing tidal restriction. We found the potential for $12 -$26M in added social benefit from 176 tons of avoided methane across 932 hectares of degraded salt marsh in Massachusetts. A significant limitation in estimating benefits, however, is the lack of coordinated, widespread monitoring strategies to infer methane and other GhGs at scale. While not insurmountable, these challenges will need to be addressed for GhG emissions reduction and/or sequestration through salt marsh restoration to be accepted as an effective strategy. We conclude that while carbon crediting may offer benefits to marsh restoration and state greenhouse gas emissions reduction targets, there remain significant limitations because of a lack of project monitoring and data validation. In the worst case, this could result in the offsetting of actual greenhouse gas emissions with credits that are supported by indirect and less-than-rigorous monitoring data.

## Introduction: Fantastic wetlands and why to monitor them

Salt marshes are critical ecosystems and are being actively restored for many reasons including habitat provision and flooding abatement, but the co-benefits of these restorations as they relate to greenhouse gas (GhG) mitigation are often overlooked. States have an opportunity to integrate methane mitigation in salt marsh restoration planning and to better catalogue these systems’ emissions reductions to contribute toward state-level GhG emissions reduction targets. To achieve these ends, better, more comprehensive monitoring and data collection is needed to inform these efforts. Once this data becomes available and more widely collected, this paper outlines a straightforward calculation to assess the GhG mitigation potential and the social benefits of salinity-increasing salt marsh restorations that could be applied at other restoration sites in Massachusetts or elsewhere. As we will discuss, the primary limitation to such an application remains data availability and lack of low-cost widespread monitoring techniques, which will be required to make this approach widely applicable and to improve validity.

Salt marshes are critical habitats that host numerous plant and animal species and provide valuable ecosystem services. Healthy salt marshes provide benefits to surrounding communities through the coastal resilience they provide against tidal surge during storms. Salt marshes also act as a carbon sink, storing a significant amount of carbon in their soils [1]. This carbon is sequestered for hundreds of years provided the marsh remains intact. However, should salt marshes become degraded or impaired, they can become a net source of GhGs rather than a sink as they can emit methane, another potent GhG [2]. One driver of excess methane emissions from salt marsh in New England is a transition from saline to fresher water in the system resulting from inadequate flushing of seawater, often due to human-induced tidal restrictions such as a narrow culvert for road/rail construction through salt marshes. Diminished salinity activates a natural microbial process that produces methane as a byproduct, thereby turning freshening salt marshes into enhanced methane sources [3, 4].

In the United States, thousands of narrow culverts that are aging or outdated impact the streams and natural areas surrounding the culvert and sometimes flood roads and other developments (MassDOT, 2020). Culvert widening, a practice used by many coastal communities’ transportation departments for road maintenance and improvement, can help to reverse the process of marsh freshening by allowing increased tidal flushing to restore salinity levels in the system, thereby reducing methane emissions. For tidal salt marsh, salinity has a negative relationship with methane emissions [4–9] and a positive relationship with carbon sequestration [9, 10] such that, as salinity increases above 18 psu (practical salinity units), methane emissions from tidal salt marsh potentially decrease and its capacity for carbon sequestration potentially increases.

Methane is 28 (100-year global warming potential) to 81 times (20-year) more potent as a greenhouse gas than carbon dioxide (CO_2_) [11]. Methane also has a shorter half-life than CO_2_ (roughly 10 years compared to 30+), so with methane mitigation, there is potential for reducing GhG effects within a shorter timeframe, possibly even within a decade. Globally, our window to act on climate change by reducing excess GhG emissions is dwindling [12]. Addressing methane emissions as quickly and from as many sources as possible, including culvert widening and other marsh restoration efforts, could afford the global community time to address more persistent sources of GhG pollution that are less susceptible to rapid remediation [13].

There are 1.7 million hectares of salt marsh throughout the United States [14], which face myriad environmental stressors; an unknown percentage of which might serve as a source of excess methane emissions due to freshening. If these marshes are to be protected or restored to mitigate rather than produce methane, more information is needed to understand the scale of benefits. These include a need to identify the spatial extent of degraded marshes, estimate the relative annual methane contributions from each marsh, identify the source of degradation (for example, a tidal restriction that prevents adequate flushing, such as a culvert), and deploy a low-cost pre- and post-restoration monitoring plan to determine the efficacy of that restoration [15]. Salinity is currently applied as a relatively cost-effective metric to track the efficacy of tidal restriction removal and in certain cases can also be used to estimate avoided methane emissions [4–6] as a result of the restoration. Though direct methane capture is a more accurate indicator of methane emissions, using salinity as a proxy for methane emissions can scope and inform policy decisions for determining prioritization of salt marsh restoration, assist in inferring social benefits of mitigated methane emissions, and potentially be used to apply for and receive carbon credits to offset a small portion of the restoration cost.

With many states working to meet mandates on GhG emissions reduction, demonstrating the potential scale of salt marsh restoration for reducing methane as a co-benefit of tidal restriction removal in salt marsh could help prioritize and expand these efforts. To demonstrate the GhG emissions reduction potential, a reliable wetland monitoring methodology will need to be implemented to track and confirm the restoration results. A standardized assessment of GhG mitigation in restored wetlands is useful not only for its contribution towards meeting environmental goals and state reduction targets, but also for the ability to count these restorations towards carbon offset crediting initiatives in addition to the ecological benefits that these projects might provide [16–20]. Provided these carbon offsets are priced appropriately, this additional revenue stream could be used to offset a portion of the cost of future salt marsh restoration projects or be used to support the long-term monitoring of restored systems. While state entities are currently barred from applying for offset credits in voluntary markets, non-profit partners are not, which could incentivize more public-private partnerships in future salt marsh restoration efforts.

This paper quantifies the potential benefits of methane reduction via salt marsh restoration at six restoration sites in Massachusetts. We estimated methane emissions reduction for each site using existing meta-analyses and approaches that use salinity as a proxy for methane emissions [4–6]. We then calculated the social benefit of these avoided emissions based on the 2021 social cost of methane [21], estimated the financial value of avoided methane emissions by applying the VCS VM00333 blue carbon crediting methodology [22]; and conclude with a discussion on current limitations and potential next steps.

## Methods

### Site selection and salinity values

We identified six completed salt marsh restoration projects administered by the Massachusetts Audubon Society (see S2 Data) that were a part of a decades-long research partnership with local middle schools in Massachusetts, USA. Each of the projects included in this analysis were those with one definitive restoration action to restore tidal flow (e.g. culvert widening) and with salinity data available both before and after the restoration project was implemented. The six projects are listed in Table 1, with their corresponding datasets listed in S2 Data. The values shown in Table 1 are those calculated by averaging all values provided both before and after the given restoration took place regardless of the location and depth at which those values were taken. The salinity datasets for these projects are hosted by the Massachusetts Audubon Society [23].

For the purposes of this analysis, a restoration project was considered successful only if the post-restoration salinity average surpassed 18 psu, which is the point at which a previously freshening salt marsh system is sufficiently saline to cease methane production methane emissions [4, 5]. As shown in Table 1, of the six sites included in this analysis, only the Mill Pond and Eastern Point sites met these criteria with the remaining sites excluded from further analysis. The Cedar Point and Seaview Street sites were excluded because averaged pre- and post-restoration salinity values remained below the 18 psu benchmark. The Town Farm Road and Conomo Point Road sites were excluded because both the pre- and post-restoration salinity values were above the 18 psu threshold.

None of these restoration projects were completed or monitored with the specific intention of methane mitigation or capture, but the pre- and post-restoration salinity data enables the retroactive application of monitoring data to estimate the potential changes in methane emissions.

### Estimating methane emissions rates and carbon sequestration

We estimated annual methane emission rates per square meter pre- and post-restoration using published methane emission factors inferred by salinity measurements in the manner described by Kroeger et al., 2017 [4]. For the pre-restoration values, we applied two emissions factors (EF): a geometric mean of studies resulting in a pre-restoration emissions factor of 19.4 gC-m^2^year^−1^ [6] and a true mean of 41.6 gC-m^2^year^−1^ [5] for salt marsh with salinity values less than 18 psu. For sites with an average salinity value above 18 psu, we applied an emissions factor of 0.46 gC-m^2^year^−1^ [5].

There is some evidence to suggest that salt marsh restoration projects that address salinity changes can additionally result in increased soil carbon sequestration, likely from increased water flow [24, 25]. While not included directly in this analysis, we demonstrate how carbon sequestration could be considered in a follow-up analysis in S1, S5 Tables.

### Applying the verified carbon standard

The Verified Carbon Standard (VCS) is a program managed by Verra, which developed the 2015 VCS Methodology for Tidal Wetland and Seagrass (VM0033) in partnership with Restore America’s Estuaries and Silvestrum [22]. Section 4c of the VM0033 on Tidal Wetlands validates culvert widening as an appropriate practice for credit application provided that pre- and post-restoration values are assessed using one of the approved methodologies. Section 8.1.4.1 allows for the use of proxies to estimate GhG emissions and section 8.1.4.4.3 allows for the use of published values to estimate emissions where proxies or direct capture are not available. Eqs 42 and 43 of Section 8.1.4.4.4. allow for the application of a default factor in conjunction with the 100-year global warming potential of methane (GWP-CH_4_) [22].

The 100-year GWP-CH_4_ as compared to the warming effects of CO_2_ over a 100-year timeframe is somewhat in flux. The U.S. Environmental Protection Agency recommends a GWP-CH_4_ range between 28–36 [26], whereas the 100-year GWP-CH_4_ assessed by the IPCC has varied across successive assessment reports, with the most recent providing a value of 27.2 ± 11, as shown in Table 2. For the purposes of this analysis, we applied the GWP-CH_4_ of 27.2, identified in the 2021 IPCC AR6 report to the calculation of verified carbon units (VCUs) using the VM0033 methodology described previously.

To estimate the VCUs for avoided methane emissions from salt marsh, as shown in Eq 1, annual post-restoration emissions were subtracted from annual pre-restoration emissions to calculate total avoided annual emissions from each site, assuming the effects of each restoration can be applied equally throughout the area of the marsh. These emissions were converted to carbon dioxide emissions (CO_2_e) by applying the average global warming potential (GWP) of methane as defined by IPCC AR6 [27], and then equated to VCUs using a 1:1 ratio (one metric ton of CO_2_e is equivalent to one VCU). Consistent with the IPCC AR6 report, we applied a GWP-CH_4_ of 27.2. Our findings are summarized in Table 3, where VCUs-yr^−1^ are equivalent to annual methane emissions avoided (as CO_2_e).

Therefore, VCUs that could have been generated at the completion of each salt marsh restoration project were estimated using the following equation:

(1) $$\text{VCU}\;=\left[E_{0}-E_{1}\right]*A*{\text{GWP}}_{\text{CH}4}+\left[C_{0}-C_{1}\right]*A$$

Where:

*VCU*—Number of Verified Carbon Unit credits (equivalent to one metric ton of emissions avoided) per year

***E* –**Emissions (metric tons of methane per square-meter) pre (_0_) and post (_1_) restoration

*A* –Area (m^2^) of restoration

*GWP*_*CH*4_ - 27.2 the global warming potential of methane (IPCC AR6)

*C*–Additional CO_2_e variables (i.e., soil carbon, nitrous oxide, etc.) pre (_0_) and post (_1_) restoration

### Calculating social benefit of avoided methane emissions and increased carbon sequestration

To estimate the social benefit of avoided GhG emissions, we applied the 2021 Social Cost of Methane [21] to emissions potentially avoided through prior wetland restoration, shown in Eq 2. An analysis of the impact of improved carbon sequestration and its associated social benefits is included in S5 Table. In this analysis, we calculate the social benefits of each salt marsh restoration as avoided costs with respect to avoided methane emissions and increased carbon sequestration.


(2) $${\text{SCM}}_{\text{2050}}=\sum_{\text{i}=2021}^{2050}\left[E_{0}-E_{1}\right]*A*Y_{i,a\%}$$

Where:

*SCM*_2050_ = Cumulative avoided social cost of methane from 2021–2050

*E* = Emissions (metric tons per square-meter) pre (_0_) and post (_1_) restoration

*Y*_*i*,*a*%_ = Social cost of methane value for year *i* discounted at *a%* described in the Interagency Working Group SCM technical document addendum (here, *a* is equivalent to 3%)

VCUs are presented in per-year values to agree with the crediting-system logic, while we integrate the social cost values over a time window (2021–2050) to account for the changes in social value over the course of the time window, based on the modeled social damages and discount rate. Values from Eq 2 are reported in Table 4 in 2021 dollars.

In February of 2021, a U.S. Government Interagency Working Group [21] proposed an update to the 2010 Social Cost of Carbon [28], and 2016 addendum to the Social Costs of Carbon (SCC) and Methane [29]. We used these updated Social Cost of Methane (SCM) values in estimating the benefits of avoided future methane emissions. We applied these updated annualized values to each of the sites included in this analysis to quantify the total potential benefit at each site realized from 2021–2050. A truncated list of annualized values is reported in Table 4 in 2021 dollars, with the full tables included in S3, S4 Tables.

Because each restoration included in this analysis occurred prior to the 2021 technical documentation or the 2016 SCM and SCC technical documents that preceded it, we were unable to calculate the total benefit of each restoration project from their implementation using the provided annualized data values. Due to this temporal inconsistency, the benefits of avoided emissions at each site were assessed from 2021 to 2050 to demonstrate the future added value from the previously completed restoration projects.

A range of potential benefits are provided using varied discount rates. Both the SCC and SCM technical documents account for uncertainty by including a range of discount rates (2.5%, 3%, and 5%). Discount rates reflect varying assumptions of the degree to which future scenarios impact current decision-making practices. The higher the discount rate, the higher the assumed value of present-day damages compared against future damages, and the lower the total social cost [28]. To keep consistent with both the 2010 SCC technical document and the 2016 SCM addendum, a three percent discount rate was treated as the default reported value; however, the full range of values is included in S3, S4 Tables.

## Results

Our analysis yielded an average annual avoided methane emissions rate of 0.19 MtCH_4_-hectare-year^−1^ and 0.41 MtCH_4_-hectare-year^−1^) for the geometric mean and true mean emissions factors, respectively. We excluded the Cedar Point, Town Farm Road, and Seaview Street sites, which did not demonstrate any avoided emissions. For the remaining Eastern Point and Mill Pond sites, we calculated the total range of avoided methane emissions from 36 MtCH4−79 MtCH_4_ (17.3 hectares total), which has the equivalent global warming potential of 985 MtCO_2_e – 2,140 MtCO_2_e. The total post-restoration monitoring periods for the Eastern Point and Mill Pond where average salinity remained above the 18 psu threshold were 12 and 11 years, respectively (see S7 Table).

### Estimating VCU accumulation

We estimated an annual VCU accumulation ranging from 5.2 VCU-hectare-year^−1^ to 11.2 VCU-hectare-year^−1^ when applying the geometric mean and true mean emissions factors, respectively. Carrying this through to the complete post-restoration monitoring periods for Eastern Point and Mill Pond sites where average annual salinity remained above 18 psu (totaling 12 and 11 years, respectively), the cumulative VCU accumulation over this time ranges from 985 VCUs to 2,140 VCUs from avoided methane alone. This translates to a potential revenue generation ranging from $19,700 to $42,800 (assuming a market price of $20/metric tonCO_2_e) when applied to the emissions factors scenarios previously described. Were these projects to have occurred today (2021), a potential revenue of 2,671 credits ($53,420) to 5,802 credits ($116,040) might have been generated between 2021–2050 across both sites (17.3 hectares), assuming that these restoration projects remain successful and consistent with the project validation requirements described by VM0033 [22], and assuming a rate of $20/MtCO_2_e. While this is a modest benefit, methane abatement is one of many associated with salt marsh restoration. It is also worth noting that whereas the social cost of carbon is discounted at a rate of 3%, we did not discount the VCS monetary values.

### The social benefit of methane mitigation

Using a 3% discount rate, we estimate a cumulative social benefit ranging from $223,253 (EF of 19.4) to $484,931 (EF of 41.6) by 2050 across the two sites (17.3 hectares) included in this analysis, as shown in Table 4.

### The big picture

McGarigal et al. (2017) mapped tidal restrictions and provided each restriction with a score from 0 (no effect) to 1 (severe effect) corresponding with an estimate of the proportion of salt marsh lost. Based on this dataset, there are potentially 475 salt marshes (932 hectares) throughout Massachusetts with tidal restrictions with an effect greater than 50% [30]. At its maximum, the successful remediation of these tidal restrictions could result in the net abatement of 176.6 to 384 MtCH_4_-year^−1^, and the potential revenue generation of $114,720 to $227,328-year^−1^ via carbon offset crediting, assuming $20/Mt-CO_2_e and a discount rate of 3%. By 2050, the social benefit of this avoided methane could range from $12 - $26 million if all sites were successfully restored, as shown in Table 4. However, the successful restoration of all sites is improbable. Of the six projects we identified in this study, only 33% - 66% achieved the types of salinity changes that would be indicative of methane abatement (depending on how salinity values are calculated). As such, this estimate likely demonstrates an extreme upper bound.

## Discussion

Dreyfus et al. (2022) describe the focus of short- to long-term greenhouse gas mitigation goals as focused on carbon dioxide with not enough attention on more non-CO_2_ pollutants like methane or nitrous oxide, which are far more potent and less controlled, though less ubiquitous [31]. In this study, we demonstrated how salt marsh restoration by way of tidal restriction removal could contribute towards the mitigation of a portion of these emissions while also increasing the resilience of these critical ecosystems, which have additional benefits such as wave attenuation, erosion control, and the provision of habitat to myriad species. There are more than 123,000 salt marshes in the United States representing 1.7 million acres of habitat, which is 31% of the global total [32]–a portion of which are likely affected or threatened by freshening. To give these systems the best chance at adaptation, addressing salinity concerns in freshening habitat wherever possible is an important and relatively low-tech application, but affordable monitoring and reliable data to support such efforts are lacking.

### More data is needed

The estimates produced in this analysis illustrate how an additional benefit of these systems might be applied once this data becomes available and should not be applied to policy decisions in their current form. Rather, this analysis was an attempt to demonstrate a road map for how such a future valuation might be possible with the appropriate data inputs. Before such an approach can be taken, better, more complete monitoring data is necessary and, to our knowledge and after extensive efforts to attain more, does not yet exist. The best salinity data that we were able to find was a longitudinal study performed by the Massachusetts Audubon Society over decades as part of an educational initiative in partnership with local middle schools. Of the sites included in this dataset, only six were associated with one definitive restoration project with both pre- and post-restoration salinity data available. That the best data currently available are those collected by middle schoolers (which is not meant to minimize the students’ contribution to the field) serves to highlight both the significant need for better data sources and the ease with which these data could be collected at sufficient scale.

### Standardized methodologies

There is more than one method to summarize salinity. The decision to average pre- and post-salinity values was only one of several ways that salinity data could have been processed for this analysis. Rather than calculate a pre- and post-restoration mean, for example, we might have also assessed these values with more fine-grained detail by examining the variance in pre-and post-restoration salinity values at different depths.

As demonstrated in field data collection worksheets prepared by the Massachusetts Audubon Society [33], salinity measurements were taken across multiple transects at each site at varying depths of “Shallow” (5 – 20cm), “Medium” (35 – 50cm), and “Deep” (65 – 80cm), these values were then aggregated, as shown in S6 Table. If depth is included as a factor in this analysis, then four of the six sites would instead be considered for further analysis. As shown in S6 Table, the first 20cm of soil in the Seaview Street and Conomo Point sites meet the previously described criteria for inclusion, in addition to both Gloucester sites previously included. Because the microbes responsible for methanogenesis are ubiquitous throughout salt marsh soil, we potentially could assume that, in these cases, methane is produced in the shallow layer among these sites and not produced in the layers of soil when salinity remains above the threshold. It is unclear whether the analyses applied by Kroeger et al. (2017) [4] accounted for these differences by depth, so this might be an avenue for further study.

Because the salinity datasets used in this paper are intended only to demonstrate the application of a valuation methodology to be applied in future restoration projects (ideally with better data), and because these datasets are middling in their quality, we chose a simplified approach by taking only the annual mean of pre-and post-restoration salinity, ignoring variance across depth or transect. We then multiplied the estimated annual pre-restoration emissions for each site by the total number of years that each post-restoration site remained above 18 psu to estimate the total avoided emissions resulting from each restoration effort within the period where salinity data is available. This methodology is oversimplified and future application of the methodology described in this paper should likely include consideration of variance by depth, time, and spatial distribution. In time, and provided that the reimbursement rate is increased from $20-ton^−1^, some of the potential revenue from VCS crediting could be used to offset some of the costs. There is a tradeoff between the accuracy of the information that can be derived from a monitoring program and the costs for implementing that monitoring program.

### The voluntary market is important but needs tightening

A market-based approach shows potential to mitigate greenhouse gas emissions, but concerns remain in the current use of salinity data to achieve those ends, especially given the recent issues demonstrated by the over-crediting of forestry credits by the California Air and Resource Board described by Badgley et al. (2021) [34] and Song and Temple (2021) [35]. While the current literature (and therefore, the VCS methodology) allows for the use of salinity data as a proxy for methane emissions from salt marsh, the application of this proxy is limited only to a range of potential emission values based on its distance from the benchmark of 18 psu. Eqs 42 and 43 of the VCS VM0033 seem to compensate for this in recommending more conservative post-restoration emissions factors of 1.1 g CH_4_-m^−2^y^−1^ for wetlands with salinity values greater than 18 psu but less than 20 psu, and 0.56 g CH_4_-m^−2^y^−1^ for salinity values greater than 20 psu compared against the post-restoration emissions factor of 0.46 as applied by Kroeger et al. (2017) [4]. Methods for the assessment of the pre-restoration emission factor are not described and therefore dependent on present-day recommendations in the literature. One concern with this approach is that the use of these benchmarks might lead to a higher than acceptable level of inaccuracy that could be avoided if the salinity approach were replaced with more methane-specific monitoring approaches. Given the importance of calculating estimated avoided emissions to potentially inform state-level GhG emissions targets and/or offset the emissions of fossil fuel-intensive activities, the development of more sensitive methods would mitigate the potential pitfalls of blue carbon crediting before it grows in popularity.

### Current crediting rates are insufficient

Our approach calculated the upper bound of potential income from all impaired salt marsh in Massachusetts, which is inclusive of 932 hectares (2,304 acres) of salt marsh and could result in the return of approximately $96,072-yr^−1^ or $42 -acre-yr^−1^ in blue carbon credits assuming $20-ton^−1^ of CO_2_e avoided. Even when applying the 20-year GWP of methane (80.8), this return only increases to $285,389-yr^−1^ ($124-acre-yr^−1^) at $20-ton^−1^ of CO_2_e avoided. This is insufficient.

While costs associated with salt marsh restoration projects (such as culvert widening or the removal of tidal restrictions) are highly variable and dependent on location, a helpful frame of reference can be found in a March 2024 grant opportunity offered by the Massachusetts Division of Ecological Restoration’s Department of Fish and Game [36], which provided grant funding ranging between $25,000 - $400,000 per project to replace aging culverts. If we use this range as a stand-in for the cost of culvert replacements in salt marsh, the scale of these potential financial returns via a voluntary blue carbon crediting program is not yet sufficient on its own to encourage additional investment in restoration, or impactful enough to meaningfully offset the cost of a restoration or even the cost of monitoring the impact of that restoration.

### An amended approach to blue carbon crediting

Currently, the VCS allows for several approaches to estimate avoided emissions: salinity as proxy, direct methane capture, or new methods supported by the literature. Because more sensitive measurements of methane emissions from degraded wetlands can be costlier and more time consuming, such approaches might be combined with cheaper options to better inform where more sensitive testing might be appropriate. For example, given the ease with which salinity assessments or remote sensing applications could be deployed (once further developed), these assessments could become common practice to assess wider swathes of salt marsh more regularly. In areas where these assessments suggest that a given restoration project was successful at mitigating GhG, (i.e., salinity values of greater than 18 psu or remote sensing instruments that detect flora consistent with higher soil salinity), more sensitive, direct capture tests could then be deployed to confirm their findings, and these more sensitive tests would then inform the allocation of carbon offset credits. If proven effective on a wider scale, newer, more innovative and low-cost approaches could be deployed to increase data collection and validity, such as the use of salt marsh flora as a salinity proxy [37, 38], or remote sensing technologies used to infer soil salinity conditions [39].

### Spatial distribution of findings remains an open question

Salt marsh methane fluxes are dynamic and can be influenced by numerous variables that include the level of the water table, temperature, and salinity, among others [40]. Further, methane emissions are not uniform across the entirety of a given salt marsh. Yet, for the purposes of this study, annual methane emissions were estimated from three discrete transects based on the limitations of the dataset and applied across the entirety of the corresponding marsh border. This is not necessarily representative of methane emissions or abatement across the whole salt marsh, yet the current VCS VM0033 methodology suggests that this approach would be valid for blue carbon offset crediting. This demonstrates another area in which the scientific literature and the VCS methodology will need to improve. Even in the case of more sensitive monitoring approaches, extrapolation of direct methane capture in a representative sample would result in a similar degree of error. Short of placing a fume hood over the entirety of a salt marsh, a degree of error will likely always exist; however, a better understanding of dynamic methane fluxes in salt marsh and the application of a representative direct capture sample with these considerations could work to reduce this error.

### The importance of getting this right

Beyond their methane fluxes, the boundaries of salt marsh systems are constantly changing in response to numerous stressors, including anthropogenic development, sea-level rise, and more. Upper marsh boundaries become lower marsh boundaries, lower marsh boundaries transition into seagrasses; and where possible as sea-levels rise, marsh extent migrates, grows, and recedes–none of these transitory properties are captured in this analysis and for our purposes, salt marsh extent was assumed to be fixed. Though many of these stressors are beyond our immediate control, salinity might be among the easiest stressors to address, thereby increasing marshes’ resilience against stressors that are less easily alleviated. Aiming to restore salinity will not work in all cases, as evidenced by the proportion of restorations that increased salinity from the data we found (2 out of6 of the Massachusetts sites). The methodologies that we applied here demonstrated moderate success in estimating the social benefits of salt marsh restoration through avoided GhG emission but were less successful in estimating the financial benefits of restoration via a voluntary carbon crediting approach. Until we have both a better understanding of salinity changes, access to more reliable salinity data, and other site-level GhG abatement measures, this approach alone is not sufficient for the distribution of blue carbon credits via a voluntary carbon market. However, this is not to say that the approach is without promise.

Salt marsh restoration is expensive and can be regulatorily complex, which can add additional associated costs, but blue carbon crediting could one day be sufficient to meaningfully offset a portion of the total restoration cost or support long-term monitoring to ensure the restoration remains effective. Until a carbon crediting system is further improved, an interim low-cost monitoring approach focused on GhG emissions reduction could still be beneficial at the state level.

The repair of tidal restrictions (undersized culverts, tide gates, dikes, or other flow restricting structures [30] is already a common, though regulatorily complex, practice among municipal and state departments of transportation for reasons other than greenhouse gas mitigation. However, including GhG mitigation potential into a State’s triaging decisions for tidal restriction repair, could be beneficial. Provided that methane data or salinity data is available, states could potentially incorporate this approach to make a first pass estimate as to the amount of methane that could be avoided as the result of a given tidal restriction restoration project; and to additionally estimate the social value added because of those avoided emissions. Such an approach might one day inform a state or municipality’s decision to repair an existing culvert with one of similar type, or to install a wider culvert to better capture the additional long-term benefits on the surrounding salt marsh and the communities they serve through the added benefits of GhG mitigation. Our assessment of salt marsh benefits is not meant to be exhaustive. This analysis covers the benefits of methane emission reduction, but other considerable benefits could include carbon sequestration.

## Conclusion

Salt marsh restoration has the potential to reduce greenhouse gas emissions, but more work needs to be done on estimating the site-level GhG abatement from restoration projects before such an approach can be reliably used to allocate voluntary carbon credits. Salt marshes offer a host of ecosystem service benefits that are not captured in this analysis. Rather, this assessment was limited only to the social benefits associated with avoided methane emissions in restored salt marsh. As this retrospective analysis has shown, standardized data for common metrics such as salinity are not readily available or regularly monitored in a way that is easily comparable. Where data does exist, it’s unclear to what degree such data can be applied to the marsh it represents–whether to a portion of the marsh area or to its entirety. And yet, this approach is currently acceptable for use in validating the success or failure of a salt marsh restoration project and could result in the assignment of VCUs to be sold in existing voluntary carbon markets. In the worst case, this could result in the offsetting of very real CO_2_e emissions with credits that are supported by less-than-rigorous monitoring data and validation processes, as has been the case in the misallocation of forestry credits [34, 35]. Carbon markets could make a significant impact in stemming the flow of future emissions, especially when considering the number of private companies who have demonstrated interest in boosting their “green” credentials, but proper allocation of credits is critical. To ensure proper allocation means the creation of effective accounting strategies and cataloguing crediting projects in a way that is verifiable and subject to regular auditing by a capable and well-resourced auditing body. If such a validation methodology were to be completed, increased trust in carbon markets could further enable nuanced applications such as the stacking of methane abatement credits with carbon sequestration and resiliency credits to better capture the plurality of benefits that salt marshes provide.

## Supplementary Material

Supplement1 **S1 Text. Supporting information text** [24, 30, 41].
(DOCX)
**S1 Table. Projected social benefits of avoided carbon 2021–2050.** Shown here are the summed annual values at each site using the social cost of carbon. The Essex and both Gloucester restoration projects were successful in increasing salinity above the 18 psu threshold, which is the level assumed to stop production of methane from impaired salt marsh. The social cost of carbon was applied to the avoided emissions at each site as if the restoration projects were completed in 2021. In contrast, the Rockport and both Ipswich restorations were not successful at restoring salinity values above the 18 psu threshold and were therefore excluded from further analysis; and the Conomo Point rd. site presented with a pre-restoration salinity value more than 18 psu. The SCC values shown here are estimates of the social damage that would have been incurred between 2021–2050 from the lower sequestration rate of carbon from these sites if no restoration had taken place, again assuming that the project was completed in 2021. The “all restricted salt marsh in MA” row demonstrates the estimated social benefits of increased carbon sequestration assuming that all sites were successfully remediated and is therefore an overestimation of the possible. This is a truncated table; the remaining table is shown in S5 Table.
(DOCX)
**S2 Table. Annual emissions savings from avoided methane and increased carbon sequestration.** This table summarizes values at each site calculated using Eq 1, where E are the annual emissions avoided, GWP is the global warming potential of methane, C is the increase in soil carbon sequestration, and VCUS-yr are the annual verified carbon units that could have been generated considering the CO_2_e potential of both avoided methane and increased carbon sequestered as a result of each restoration assuming that the project was considered for carbon credits via the Verified Carbon Standard.
(DOCX)
**S3 Table. Social benefit of methane using an EF of 19.4.** The social benefit of Methane for the two sites included in the study, in addition to the 475 (932 hectares) of Massachusetts salt marshes impaired by tidal restrictions, as determined by McGarigal et al., 2017. Benefits calculated with SCM values using an EF of 19.4. Values are shown in $2021.
(XLSX)
**S4 Table. Social benefits of methane using an EF of 41.6.** The social benefit of Methane applied to both sites included in the study, in addition to the 475 (932 hectares) of Massachusetts salt marshes impaired by tidal restrictions, as determined by McGarigal et al., 2017. Benefits calculated with SCM values using an EF of 41.6. Values are shown in $2021.
(XLSX)
**S5 Table. Social benefit of carbon.** The social benefit of Carbon applied to both sites included in the study, in addition to the 475 (932 hectares) of Massachusetts salt marshes impaired by tidal restrictions, as determined by McGarigal et al., 2017. Benefits calculated with SCC values. Values do not vary by EF so EF excluded. Values are shown in $2021.
(XLSX)
**S6 Table. Restored marsh salinity variance by depth.** Shown here is the variance in pre-and post-restoration salinity values with associated standard error by degrees of depth. As described by the Massachusetts Audubon Society, salinity values were measured at discrete depths of Shallow” (5 – 20cm), “Medium” (35– 50cm), and “Deep” (65 – 80cm). When average pre- and post-restoration salinity is corrected by depth, we can see a much stronger change in the first 20cms of soil compared against the remaining 60cms. (DOCX)
**S7 Table. Restored marsh salinity variance by year.** Of the six sites included in this analysis, shown here is the annual variance in salinity values demonstrated both before and after the first restoration year, which is demarcated by the grey highlighted cell for each site. Post-restoration sites that were below 18 ppt are demarcated by the orange highlighted cell and were not included in our estimates of avoided methane emissions since methane production during these years was possible. As is shown, average annual salinity values vary year-on-year both before and after implementation of the restoration project at each site. Further, this summation demonstrates a definitive lag phase in the Gloucester sites following the completed restoration project. Were this lag phase to have been included in our overall analysis, the total social benefit value for each site would have been reduced to only include years in which the average salinity was above 18 psu. Average pre- and post-salinity values vary here when compared against previous summary tables as these averages represent an *average of the annual averages* rather than an average of the pre- and post-restoration values.
(DOCX)
**S1 Data. This Excel model was developed by the authors to determine the social costs and benefits of carbon and methane release/avoidance at each of the salt marsh sites considered for this analysis.** The model can be manipulated by changed the input for emissions factor (EF).
(XLSX)
S2 Data. Salinity data sources from the Massachusetts Audubon society.
(DOCX)

## Figures and Tables

**Table 1.** Restored marsh name, location, size, salinity values. A summary table for the six sites selected for analysis from the Massachusetts Audubon Society dataset. Each site includes pre- and post-restoration salinity data with associated standard error at varying times and seasons. Due to this variance, pre- and post-salinity values were averaged without taking temporal considerations into account. Total marsh area for the Essex site was derived from MA CZM records, the Town Farm Road area was obtained by Mass.gov, Seaview Street was derived from NO1 records, Mill Pond was derived from U.S. Fish and Wildlife data. The remaining sites were derived from MA DEP records.

Location	Years Monitored	Date of restoration	Years of Post-restoration Monitoring	Number of Data Points	Avg Salinity pre-rest (ppt)	Avg Salinity post-rest (ppt)	Total Area of Wetland (m^2)	Total Area of Wetland (hectares)
Essex, MA, Conomo Point Road	1998–2015	Tidal Restriction restored November 2000	15	961	18.3 ± 0.94	23.7 ± 0.41	54,197	5.42
Gloucester, MA, Eastern Point	2000–2015	Tidal Restriction restored November 2003	12	764	10.2 ± 0.52	19 ± 0.3	10,955	1.10
Ipswich, MA, Cedar Point	1999–2015	Tidal Restriction Phase 1 restored Spring 2000	15	249	12.6 ± 0.75	9.8 ± 0.63	12,869	1.29
Ipswich, MA, Town Farm Road	1996–2015	Tidal Restriction restored spring 2005	10	829	27.4 ± 0.51	25.7 ± 0.36	97,128	9.71
Rockport, MA Seaview Street	1998–2015	Seaview Street tidal restriction restored fall 2003	12	371	17.7 ± 1.7	16.9 ± 0.42	12,141	1.21
Gloucester, MA, Mill Pond	1998–2015	Tidal Restriction -Site flooded when board placed on tide gates spring 2003, killing marsh plants. Tide gates opened beginning spring 2004	11	808	17.4 ± 0.44	19.7 ± 0.32	161,880	16.19

**Table 2.** Change in IPCC global warming potentials. Change in IPCC global warming potentials (GWP) for methane (CH_4_) and nitrous oxide (N_2_O) across assessment reports (AR) 4, 5, and 6.

		100-year GWP			20-year GWP		
Greenhouse Gas (GhG)	Lifetime^a^ (years)	AR4 (2007)	AR5 (2014)	AR6 (2021)	AR4 (2007)	AR5 (2014)	AR6 (2021)
CO_2_	Multiple	1	1	1	1	1	1
CH_4_ (non-fossil)	11.8 ± 1.8	25	28^b^–34^c^	27.2 ± 11^d^	21	84^b^–86^c^	80.8 ± 25.8^d^
N_2_O	109 ± 10	298	265^b^ –298^c^	273 ± 130^d^	310	264^b^-268^c^	273 ± 118^d^

a Compound lifetime values were reported in IPCC AR6.

b Non-inclusive of climate change feedback, as reported in AR5

c Inclusive of climate change feedback

d AR6 was the only report to include error ranges

**Table 3.** Summary of annual avoided emissions. Summary of values from each site calculated using Eq 1, where E are the annual emissions avoided, GWP is the global warming potential of methane, and VCUS-yr are the annual verified carbon units that could have been generated considering the CO_2_e potential of both avoided methane and increased carbon sequestered as a result of each restoration assuming that the project was considered for carbon credits via the Verified Carbon Standard. Values are shown using both emissions factors (EF) of 19.4 (geometric mean) and 41.6 (true mean) to demonstrate range of values.

EF 19.4 EF 41.6	Eastern Point	Mill Pond	Total
E-yr^−1^	0.21 0.45	3.07 6.66	3.28 7.11
GWP_CH4_	27.2	27.2	27.2
VCUs-yr^−1^	5.7 12.2	83.5 181.2	89.2 193.4

**Table 4.** Social benefit value of avoided methane emissions. The social benefit value of avoided methane applied to the two sites included in the study, in addition to the 475 (932 hectares) of Massachusetts salt marshes impaired by tidal restrictions, as determined by McGarigal et al. (2017) [30]. Benefits calculated with Social Cost of Methane values proposed by the 2021 Interagency Working Group [21] using emissions factors (EF) of 19.4 and 41.6. *This is a truncated table; the full table can be found in* S3, S4 Tables.

Year	Avoided social cost of methane (3% average) in 2021 dollars ($/Mt)	Gloucester, MA, Eastern Point EF 19.4 EF 41.6	Gloucester, MA, Mill Pond EF 19.4 EF 41.6	All restricted salt marsh in MA (932 hectares) EF 19.4 EF 41.6
**2021**	$1,500.00	$311 $676	$4,599 $9,990	$264,903 $575,402
**2022**	$1,600.00	$332 $721	$4,906 $10,656	$282,563 $613,762
**2023**	$1,600.00	$332 $721	$4,906 $10,656	$282,563 $613,762
**2024**	$1,700.00	$353 $766	$5,212 $11,322	$300,223 $652,122
**2025**	$1,700.00	$353 $766	$5,212 $11,322	$300,223 $652,122
**2026**	$1,800.00	$373 $811	$5,519 $11,988	$317,884 $690,482
**2027**	$1,800.00	$373 $811	$5,519 $11,988	$317,884 $690,482
**2028**	$1,900.00	$394 $856	$5,825 $12,654	$335,544 $728,842
**2029**	$1,900.00	$394 $856	$5,825 $12,654	$335,544 $728,842
**2030**	$2,000.00	$415 $901	$6,132 $13,319	$353,204 $767,202
**2021–2050 Total**	-	$14,151 $30,737	$209,102 $454,194	$12,044,257 $26,161,602

## Data Availability

All salinity data used in this analysis was collected by Massachusetts Audubon Society and archived via their website: https://www.massaudubon.org/get-outdoors/wildlife-sanctuaries/endicott/salt-marsh-project/results-data/salinity This data was processed by the authors via the Excel model provided as part of this submission.
