# Supplementary material for: Fantastic wetlands and why to monitor them: Demonstrating the social and financial benefit potential of methane abatement through salt marsh restoration
Source: PLOS Clim. Author manuscript; Available in PMC 2025 Jul 5. (PMC11457170; doi:10.1371/journal.pclm.0000317)
Supplement: Supplement1 — S1 Text. Supporting information text [24, 30, 41]. (DOCX) S1 Table. Projected social benefits of avoided carbon 2021–2050. Shown here are the summed annual values at each site using the social cost of carbon. The Essex and both Gloucester restoration projects were successful in increasing salinity above the 18 psu threshold, which is the level assumed to stop production of methane from impaired salt marsh. The social cost of carbon was applied to the avoided emissions at each site as if the restoration projects were completed in 2021. In contrast, the Rockport and both Ipswich restorations were not successful at restoring salinity values above the 18 psu threshold and were therefore excluded from further analysis; and the Conomo Point rd. site presented with a pre-restoration salinity value more than 18 psu. The SCC values shown here are estimates of the social damage that would have been incurred between 2021–2050 from the lower sequestration rate of carbon from these sites if no restoration had taken place, again assuming that the project was completed in 2021. The “all restricted salt marsh in MA” row demonstrates the estimated social benefits of increased carbon sequestration assuming that all sites were successfully remediated and is therefore an overestimation of the possible. This is a truncated table; the remaining table is shown in S5 Table. (DOCX) S2 Table. Annual emissions savings from avoided methane and increased carbon sequestration. This table summarizes values at each site calculated using Eq 1, where E are the annual emissions avoided, GWP is the global warming potential of methane, C is the increase in soil carbon sequestration, and VCUS-yr are the annual verified carbon units that could have been generated considering the CO2e potential of both avoided methane and increased carbon sequestered as a result of each restoration assuming that the project was considered for carbon credits via the Verified Carbon Standard. (DOCX) S3 Table. Social benefit [file NIHMS2015136-supplement-Supplement1.zip › pclm.0000317.s001.docx]

**SUPPORTING INFORMATION**

**LEGEND:**

**S1 Table:** Projected Social Benefits of Avoided Carbon 2021-2050.

**S2 Table:** Annual emissions savings from avoided methane and increased carbon sequestration.

**S3 Table:** Social benefit of Methane using an EF of 19.4.

**S4 Table:** Social benefits of Methane using an EF of 41.6

**S5 Table:** Social benefit of Carbon.

**S6 Table:** Restored Marsh Salinity Variance by Depth.

**S7 Table:** Restored Marsh Salinity Variance by Year.

**S1 Data:** Excel Model.

**S2 Data:** Salinity data sources.

*The Social Benefit of Carbon Sequestration in Massachusetts Marshes*

Carbon sequestration values were not measured directly in this analysis; however, it’s likely that the rate of carbon sequestration increased in areas where salt marsh restoration projects were successful. Due to limited carbon sequestration data at each of the sites included in this study, we applied a soil carbon sequestration rate of 100 gC-m^2^year^-1^ for impaired salt marsh and a 200 gC-m^2^year^-1^ for restored marsh, defined as those marshes with a post-restoration value greater than 18 psu [24][41]. Though we acknowledge that the question of carbon sequestration rates are far more complex than these benchmark values would suggest.

As explained in the main body of the paper, we identified 475 salt marshes (932 hectares) throughout Massachusetts with tidal restrictions with an effect greater than 50% as identified by McGarigal et al., 2017 [30]. The successful remediation of these tidal restrictions could result in an upper-limit^[[1]](#footnote-1)^ net abatement of 932 MtC-year^-1^, assuming all restorations resulted in an increase of system salinity above 18 psu.

We applied the social cost of carbon to this carbon abatement value using the following equation:

$${SCC}_{2050}= \sum_{i=2021}^{2050} {[E}_{0}-E_{1}]*A* Z_{i,b\%}$$

Where:

${SCC}_{2050}$ – Cumulative avoided social cost of carbon from 2021-2050

$E$ – Emissions (metric tons per square-meter) pre (0) and post (1) restoration

$A$ – Area (m^2^) of restoration

$Z_{i,b\%}$ - Social cost of carbon value for year *i* discounted at *b*% as described in the EPA SCC technical documentation (here, *b* is equivalent to either 2.5%, 3%, or 5%).

The estimated social benefits of increased carbon sequestration from saltmarsh restoration, at discount rates of 2.5%, 3%, and 5%, is presented in S1 Table.

**S1 Table:** **Projected Social Benefits of Avoided Carbon 2021-2050.** Shown here are the summed annual values at each site using the social cost of carbon. The Essex and both Gloucester restoration projects were successful in increasing salinity above the 18 psu threshold, which is the level assumed to stop production of methane from impaired salt marsh. The social cost of carbon was applied to the avoided emissions at each site as if the restoration projects were completed in 2021. In contrast, the Rockport and both Ipswich restorations were not successful at restoring salinity values above the 18 psu threshold and were therefore excluded from further analysis; and the Conomo Point rd. site presented with a pre-restoration salinity value more than 18 psu. The SCC values shown here are estimates of the social damage that would have been incurred between 2021-2050 from the lower sequestration rate of carbon from these sites if no restoration had taken place, again assuming that the project was completed in 2021. The “all restricted salt marsh in MA” row demonstrates the estimated social benefits of increased carbon sequestration assuming that all sites were successfully remediated and is therefore an overestimation of the possible. This is a truncated table; the remaining table is shown in **S5 Table**.

|  | **Projected Benefits from 2021 – 2050 using 2021 Social Cost of Carbon** | | |
| --- | --- | --- | --- |
| **Location** | **Avoided social cost of carbon by 2050 (5% average) in 2021 dollars** | **Avoided social cost of carbon by 2050 (3% average) in 2021 dollars** | **Avoided social cost of carbon by 2050 (2.5% average) in 2021 dollars** |
| **Essex, MA, Conomo Point Road** |  |  |  |
| **Gloucester, MA, Eastern Point** | $748 | $2,239 | $3,187 |
| **Ipswich, MA, Cedar Point** |  |  |  |
| **Ipswich, MA, Town Farm Road*** |  |  |  |
| **Rockport, MA Seaview Street** |  |  |  |
| **Gloucester, MA, Mill Pond** | $11,056 | $33,088 | $47,091 |
| **All restricted salt marsh in MA (932 hectares)** | $636,849 | $1,905,884 | $2,712,435 |
| **All restricted salt marsh in MA (conservative estimate, assuming an effect ≥ 50%)** | $318,425 | $952,942 | $1,356,218 |

Like the SCM analysis previously described, these estimates of social benefit value resulting from the future restoration of all tidally restricted salt marsh demonstrate an upper bound of potential social benefits given the limitations associated with salinity rebound in restored salt marsh. All caveats previously described also apply to this SCC analysis.

Using Equation 1 above, we estimated that the restoration of each tidal restriction resulted in an increased carbon sequestration of 191.2 MtCO_2_ in either EF scenario^[[2]](#footnote-2)^.

**S2 Table: Annual emissions savings from avoided methane and increased carbon sequestration**. This table summarizes values at each site calculated using Equation 1, where E are the annual emissions avoided, GWP is the global warming potential of methane, C is the increase in soil carbon sequestration, and VCUS-yr are the annual verified carbon units that could have been generated considering the CO_2_e potential of both avoided methane and increased carbon sequestered as a result of each restoration assuming that the project was considered for carbon credits via the Verified Carbon Standard.

| EF 19.4  EF 41.6 | Eastern Point | Mill Pond | Totals |
| --- | --- | --- | --- |
| E-yr^-1^ | 0.21  0.45 | 3.07  6.66 | 3.28  7.11 |
| GWP_CH4_ | 27.2 | 27.2 | 27.2 |
| C-yr^-1^ | 1.1 | 16.2 | 17.3 |
| VCUs-yr^-1^ | 6.8  13.3 | 99.7  197.4 | 106.5  210.7 |


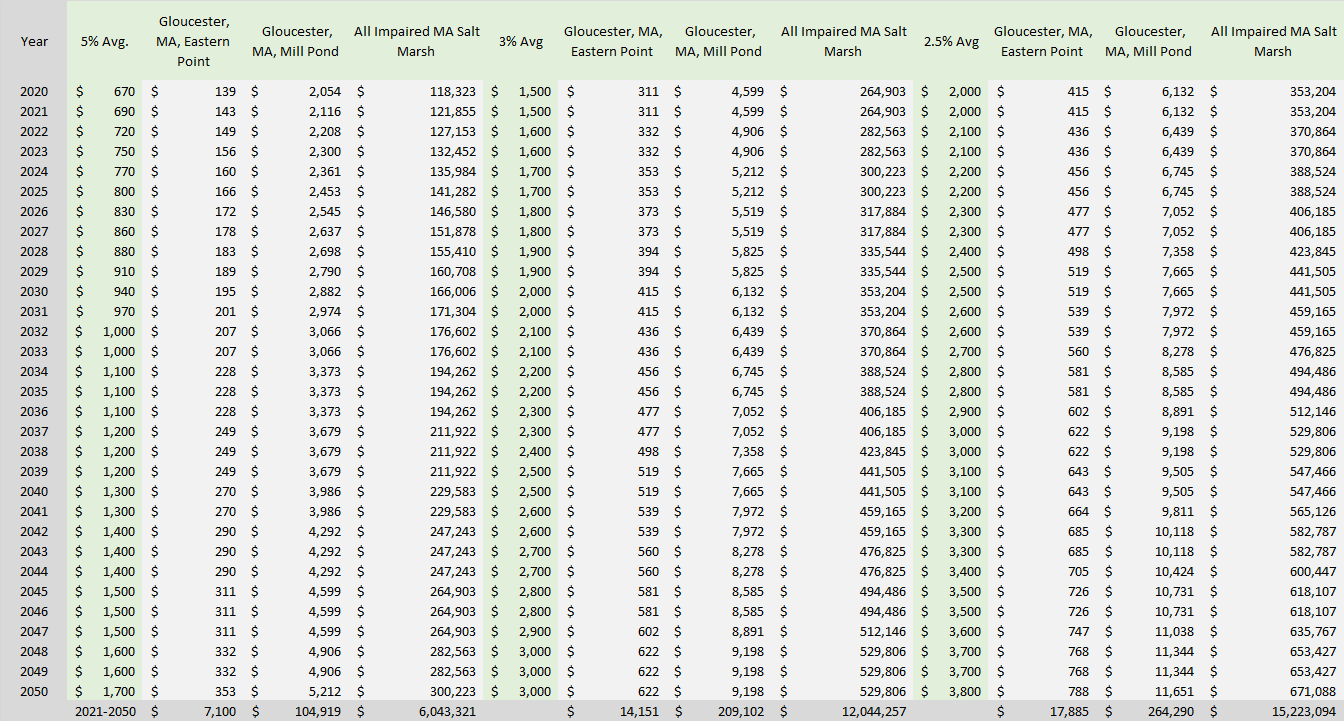


**S3 Table:** The social benefit of Methane for the two sites included in the study, in addition to the 475 (932 hectares) of Massachusetts salt marshes impaired by tidal restrictions, as determined by McGarigal et al., 2017. Benefits calculated with SCM values using an EF of 19.4. Values are shown in $2021


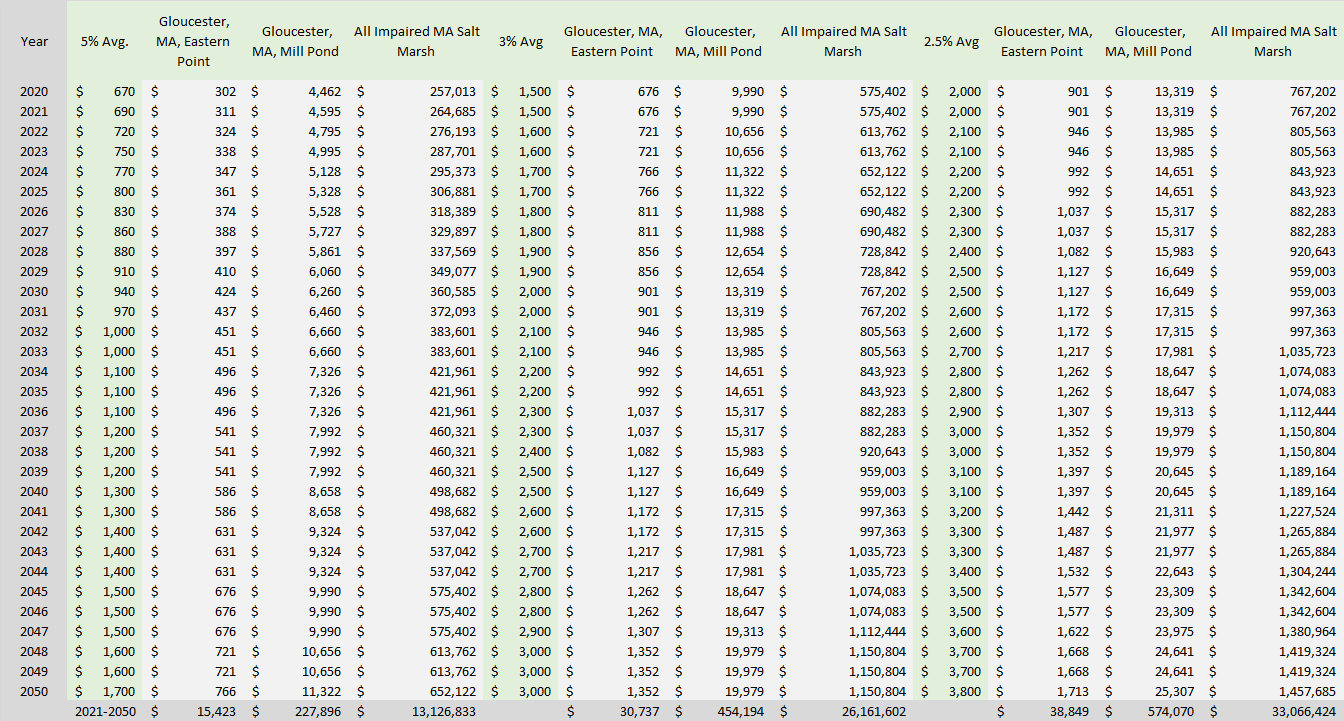


**S4 Table:** The social benefit of Methane applied to both sites included in the study, in addition to the 475 (932 hectares) of Massachusetts salt marshes impaired by tidal restrictions, as determined by McGarigal et al., 2017. Benefits calculated with SCM values using an EF of 41.6. Values are shown in $2021


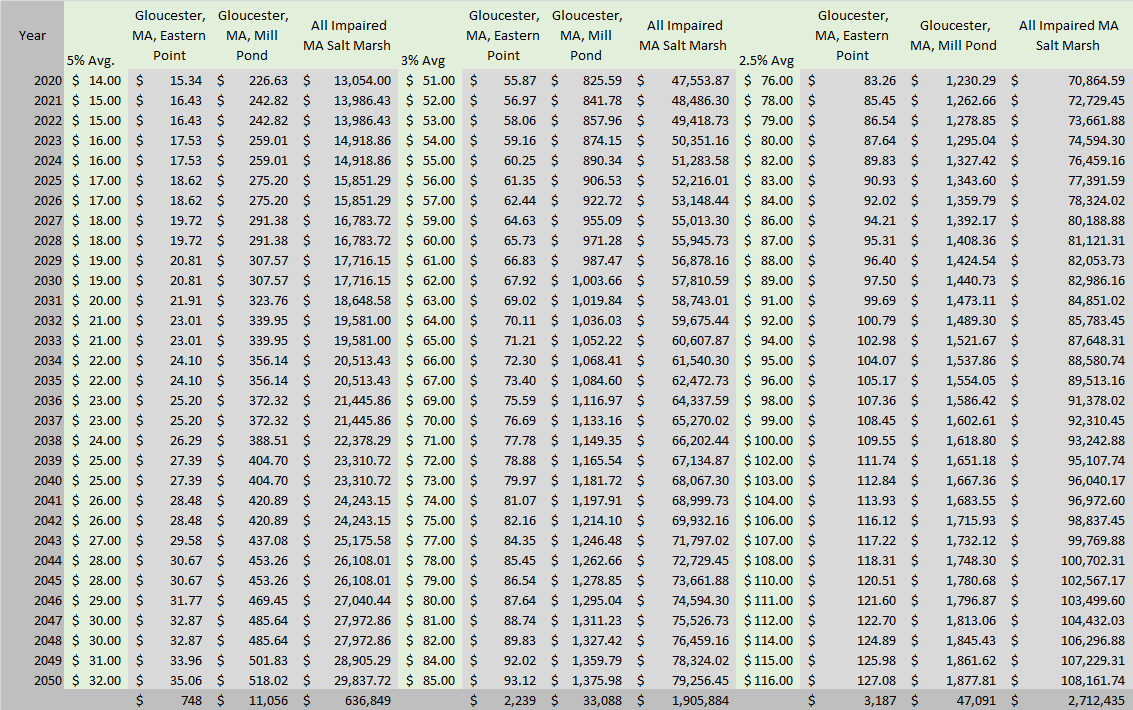


**S5 Table:** The social benefit of Carbon applied to both sites included in the study, in addition to the 475 (932 hectares) of Massachusetts salt marshes impaired by tidal restrictions, as determined by McGarigal et al., 2017. Benefits calculated with SCC values. Values do not vary by EF so EF excluded. Values are shown in $2021

**S6 Table: Restored Marsh Salinity Variance by Depth.** Shown here is the variance in pre-and post-restoration salinity values with associated standard error by degrees of depth. As described by the Massachusetts Audubon Society, salinity values were measured at discrete depths of Shallow” (5 – 20cm), “Medium” (35– 50cm), and “Deep” (65 – 80cm). When average pre- and post-restoration salinity is corrected by depth, we can see a much stronger change in the first 20cms of soil compared against the remaining 60cms.

|  |  | Shallow | Medium | Deep |
| --- | --- | --- | --- | --- |
| Essex Conomo Point | Restricted | 7.8 ± 0.54 | 23.6 ± 0.52 | 22 ± 0.52 |
|  | Restored | 22.4 ± 0.6 | 24.4 ± 0.58 | 24.2 ± 0.58 |
| Gloucester Eastern Point | Restricted | 8.1 ± 0.36 | 10.8 ± 0.34 | 11.9 ± 0.36 |
|  | Restored | 19.4 ± 0.47 | 19.2 ± 0.45 | 18.4 ± 0.48 |
| Ipswich Cedar Point | Restricted | 11.8 ± 0.39 | 12.8 ± 0.34 | 13.4 ± 0.27 |
|  | Restored | 11.7 ± 0.92 | 11.3 ± 0.79 | 8.8 ± 0.62 |
| Ipswich Town Farm | Restricted | 31 ± 0.5 | 29.3 ± 0.49 | 24.8 ± 0.49 |
|  | Restored | 28.9 ± 0.45 | 27.1 ± 0.44 | 24.8 ± 0.44 |
| Rockport Seaview St. | Restricted | 17.5 ± 0.59 | 18.2 ± 0.57 | 17.4 ± 0.56 |
|  | Restored | 20.4 ± 0.73 | 16.1 ± 0.7 | 13.9 ± 0.69 |
| *Gloucester Mill Pond* | *Restricted* | *12.4 ± 0.48* | *18.5 ± 0.47* | *21.7 ± 0.48* |
|  | *Restored* | *22.3 ± 0.44* | *18.4 ± 0.43* | *19.1 ± 0.43* |

**S7 Table: Restored Marsh Salinity Variance by Year.** Of the six sites included in this analysis, shown here is the annual variance in salinity values demonstrated both before and after the first restoration year, which is demarcated by the grey highlighted cell for each site. Post-restoration sites that were below 18 ppt are demarcated by the orange highlighted cell and were not included in our estimates of avoided methane emissions since methane production during these years was possible. As is shown, average annual salinity values vary year-on-year both before and after implementation of the restoration project at each site. Further, this summation demonstrates a definitive lag phase in the Gloucester sites following the completed restoration project. Were this lag phase to have been included in our overall analysis, the total social benefit value for each site would have been reduced to only include years in which the average salinity was above 18 psu. Average pre- and post-salinity values vary here when compared against previous summary tables as these averages represent an *average of the annual averages* rather than an average of the pre- and post-restoration values.

| ***Year*** | ***Essex Conomo Point*** | ***Gloucester Eastern Point*** | ***Gloucester Mill Pond*** |  | ***Ipswich Cedar Rd.*** | ***Ipswich Town Farm*** | ***Rockport Saratoga*** |
| --- | --- | --- | --- | --- | --- | --- | --- |
| *1996* |  |  |  |  |  | *23.8 ± 1.04* |  |
| *1997* |  |  |  |  |  | *No Data* |  |
| *1998* | *20.7 ± 1.7* |  | *21 ± 0.8* |  |  | *29 ± 0.96* | *No Data* |
| *1999* | *15.7 ± 1.39* |  | *21.7 ± 1.33* |  | *12.6 ± 0.75* | *33.6 ± 2.66* | *No Data* |
| *2000* | *20 ± 1.72* | *12.1 ± 1.32* | *18.2 ± 0.71* |  | *10 ± 1.43* | *28 ± 1.28* | *No Data* |
| *2001* | *23.4 ± 0.96* | *13.2 ± 1.22* | *14.2 ± 0.68* |  | *5.1 ± 2.05* | *26 ± 1.3* | *No Data* |
| *2002* | *21.7 ± 1.46* | *10.9 ± 0.95* | *No Data* |  | *No Data* | *29.2 ± 0.96* | *18.2 ± 1.65* |
| *2003* | *25.5 ± 1.53* | *No Data* | *No Data* |  | *No Data* | *30.7 ± 1.12* | *No Data* |
| *2004* | *20.6 ± 0.67* | *16.3 ± 0.85* | *21 ± 2.96* |  | *7.3 ± 0.67* | *21.1 ± 1.12* | *13 ± 1.41* |
| *2005* | *23.2 ± 1.04* | *20.5 ± 1.11* | *16.2 ± 0.74* |  | *11 ± 4* | *24.3 ± 1.36* | *20.2 ± 1.66* |
| *2006* | *22.1 ± 1.18* | *18.6 ± 0.59* | *15 ± 0.63* |  | *8.5 ± 1.28* | *23.6 ± 1.01* | *11.2 ± 1.31* |
| *2007* | *24 ± 1.19* | *22.3 ± 1.66* | *18.4 ± 0.58* |  | *6.9 ± 0.68* | *24.5 ± 1.03* | *16.2 ± 1.29* |
| *2008* | *22.5 ± 1.21* | *19 ± 1.45* | *18.8 ± 0.91* |  | *6.5 ± 0.81* | *23.1 ± 1.16* | *10.2 ± 2.11* |
| *2009* | *19.3 ± 1.05* | *17.3 ± 1.15* | *19.3 ± 0.93* |  | *7.5 ± 1.27* | *23.3 ± 0.99* | *15.8 ± 1.75* |
| *2010* | *21.4 ± 0.73* | *19.1 ± 1.47* | *22.5 ± 1.21* |  | *14.9 ± 1.01* | *26.7 ± 0.96* | *22.3 ± 1.3* |
| *2011* | *19.4 ± 0.95* | *16.3 ± 1.59* | *20.7 ± 1.18* |  | *No Data* | *25.5 ± 1.2* | *12.9 ± 1.42* |
| *2012* | *24.6 ± 0.72* | *19.2 ± 1.26* | *16.5 ± 1.32* |  | *15.6 ± 1.32* | *28.4 ± 1.49* | *13.3 ± 0.98* |
| *2013* | *20.9 ± 0.8* | *20.4 ± 2.18* | *18.6 ± 1.04* |  | *10.9 ± 1.43* | *25.7 ± 0.95* | *17.7 ± 1.07* |
| *2014* | *25.8 ± 0.94* | *17.8 ± 1.77* | *22.6 ± 1.23* |  | *11.2 ± 4.23* | *24.5 ± 0.73* |  |
| *2015* | *29.1 ± 4.02* | *19.5 ± 1.84* | *25.1 ± 1.32* |  | *9.9 ± 1.44* | *25.9* |  |
| *2016* |  | *20 ± 1.28* | *29.9 ± 0.99* |  | *6.8 ± 1.23* | *29.8* |  |
| *2017* |  | *20.8 ± 1* | *25.1 ± 0.87* |  | *10.9 ± 1.45* | *27.3* |  |
| *2018* |  | *18.5 ± 0.63* | *21 ± 1.12* |  | *15.1 ± 1.53* | *28.2* |  |
| *Avg. Pre* | *18.2 ± 1.54* | *12.1 ± 1.16* | *18.8 ± 0.88* |  | *12.6 ± 0.75* | *27.7 ± 1.31* | *18.2 ± 1.65* |
| *Avg. Post* | *22.9 ± 1.25* | *19.2 ± 1.36* | *20.7 ± 1.14* |  | *9.9 ± 1.63* | *25.9 ± 1.06* | *15.3 ± 1.43* |

**S1 Data:** This Excel model was developed by the authors to determine the social costs and benefits of carbon and methane release/avoidance at each of the salt marsh sites considered for this analysis. The model can be manipulated by changed the input for emissions factor (EF).

**S2 Data. Salinity data sources**

Massachusetts Audubon Society salinity results and values: <https://www.massaudubon.org/get-outdoors/wildlife-sanctuaries/endicott/salt-marsh-project/results-data/salinity>

[Essex, MA Conomo Point Rd.](https://www.massaudubon.org/content/download/9356/155752/file/essewell.zip): https://www.massaudubon.org/content/download/9356/155752/file/essewell.zip

[Gloucester, MA, Eastern Point:](https://www.massaudubon.org/content/download/9346/155712/file/glepwell.zip)  https://www.massaudubon.org/content/download/9346/155712/file/glepwell.zip

[Gloucester, MA, Mill Pond](https://www.massaudubon.org/content/download/9350/155728/file/glouwell.zip): https://www.massaudubon.org/content/download/9350/155728/file/glouwell.zip

[Ipswich, MA, Cedar Point](https://www.massaudubon.org/content/download/9375/155828/file/iptpwell.zip): https://www.massaudubon.org/content/download/9375/155828/file/iptpwell.zip

[Ipswich, MA, Town Farm Road](https://www.massaudubon.org/content/download/9358/155760/file/ips1well.zip): https://www.massaudubon.org/content/download/9358/155760/file/ips1well.zip

[Rockport, MA, Seaview Street (excluding Saratoga Creek)](https://www.massaudubon.org/content/download/9377/155836/file/rockwell.zip): https://www.massaudubon.org/content/download/9377/155836/file/rockwell.zip

1. As our previous analysis has shown, of the 6 sites included in our analysis, only 33% - 66% of these restorations were successful (depending on how salinity values are summarized). Therefore, a more apt estimate would likely be 33% - 66% of 932 MTC-year^-1^. [↑](#footnote-ref-1)
2. Because carbon sequestration was estimated using a value not dependent on an emissions factor, the carbon accretion rate remains constant across either scenario. [↑](#footnote-ref-2)
