# Supplementary material for: Fantastic wetlands and why to monitor them: Demonstrating the social and financial benefit potential of methane abatement through salt marsh restoration
Source: PLOS Clim. Author manuscript; Available in PMC 2025 Jul 5. (PMC11457170; doi:10.1371/journal.pclm.0000317)
Supplement: Supplement1 — S1 Text. Supporting information text [24, 30, 41]. (DOCX) S1 Table. Projected social benefits of avoided carbon 2021–2050. Shown here are the summed annual values at each site using the social cost of carbon. The Essex and both Gloucester restoration projects were successful in increasing salinity above the 18 psu threshold, which is the level assumed to stop production of methane from impaired salt marsh. The social cost of carbon was applied to the avoided emissions at each site as if the restoration projects were completed in 2021. In contrast, the Rockport and both Ipswich restorations were not successful at restoring salinity values above the 18 psu threshold and were therefore excluded from further analysis; and the Conomo Point rd. site presented with a pre-restoration salinity value more than 18 psu. The SCC values shown here are estimates of the social damage that would have been incurred between 2021–2050 from the lower sequestration rate of carbon from these sites if no restoration had taken place, again assuming that the project was completed in 2021. The “all restricted salt marsh in MA” row demonstrates the estimated social benefits of increased carbon sequestration assuming that all sites were successfully remediated and is therefore an overestimation of the possible. This is a truncated table; the remaining table is shown in S5 Table. (DOCX) S2 Table. Annual emissions savings from avoided methane and increased carbon sequestration. This table summarizes values at each site calculated using Eq 1, where E are the annual emissions avoided, GWP is the global warming potential of methane, C is the increase in soil carbon sequestration, and VCUS-yr are the annual verified carbon units that could have been generated considering the CO2e potential of both avoided methane and increased carbon sequestered as a result of each restoration assuming that the project was considered for carbon credits via the Verified Carbon Standard. (DOCX) S3 Table. Social benefit [file NIHMS2015136-supplement-Supplement1.zip › pclm.0000317.s002.docx]

**S2 Table: Projected Social Benefits of Avoided Carbon 2021-2050.** Shown here are the summed annual values at each site using the social cost of carbon. The Essex and both Gloucester restoration projects were successful in increasing salinity above the 18 psu threshold, which is the level assumed to stop production of methane from impaired salt marsh. The social cost of carbon was applied to the avoided emissions at each site as if the restoration projects were completed in 2021. In contrast, the Rockport and both Ipswich restorations were not successful at restoring salinity values above the 18 psu threshold and were therefore excluded from further analysis; and the Conomo Point rd. site presented with a pre-restoration salinity value more than 18 psu. The SCC values shown here are estimates of the social damage that would have been incurred between 2021-2050 from the lower sequestration rate of carbon from these sites if no restoration had taken place, again assuming that the project was completed in 2021. The “all restricted salt marsh in MA” row demonstrates the estimated social benefits of increased carbon sequestration assuming that all sites were successfully remediated and is therefore an overestimation of the possible. This is a truncated table; the remaining table is shown in Appendix B.

|  | **Projected Benefits from 2021 – 2050 using 2021 Social Cost of Carbon** | | |
| --- | --- | --- | --- |
| **Location** | **Avoided social cost of carbon by 2050 (5% average) in 2021 dollars** | **Avoided social cost of carbon by 2050 (3% average) in 2021 dollars** | **Avoided social cost of carbon by 2050 (2.5% average) in 2021 dollars** |
| **Essex, MA, Conomo Point Road** |  |  |  |
| **Gloucester, MA, Eastern Point** | $748 | $2,239 | $3,187 |
| **Ipswich, MA, Cedar Point** |  |  |  |
| **Ipswich, MA, Town Farm Road*** |  |  |  |
| **Rockport, MA Seaview Street** |  |  |  |
| **Gloucester, MA, Mill Pond** | $11,056 | $33,088 | $47,091 |
| **All restricted salt marsh in MA (932 hectares)** | $636,849 | $1,905,884 | $2,712,435 |
| **All restricted salt marsh in MA (conservative estimate, assuming an effect ≥ 50%)** | $318,425 | $952,942 | $1,356,218 |
