# Supplementary material for: Fantastic wetlands and why to monitor them: Demonstrating the social and financial benefit potential of methane abatement through salt marsh restoration
Source: PLOS Clim. Author manuscript; Available in PMC 2025 Jul 5. (PMC11457170; doi:10.1371/journal.pclm.0000317)
Supplement: Supplement1 — S1 Text. Supporting information text [24, 30, 41]. (DOCX) S1 Table. Projected social benefits of avoided carbon 2021–2050. Shown here are the summed annual values at each site using the social cost of carbon. The Essex and both Gloucester restoration projects were successful in increasing salinity above the 18 psu threshold, which is the level assumed to stop production of methane from impaired salt marsh. The social cost of carbon was applied to the avoided emissions at each site as if the restoration projects were completed in 2021. In contrast, the Rockport and both Ipswich restorations were not successful at restoring salinity values above the 18 psu threshold and were therefore excluded from further analysis; and the Conomo Point rd. site presented with a pre-restoration salinity value more than 18 psu. The SCC values shown here are estimates of the social damage that would have been incurred between 2021–2050 from the lower sequestration rate of carbon from these sites if no restoration had taken place, again assuming that the project was completed in 2021. The “all restricted salt marsh in MA” row demonstrates the estimated social benefits of increased carbon sequestration assuming that all sites were successfully remediated and is therefore an overestimation of the possible. This is a truncated table; the remaining table is shown in S5 Table. (DOCX) S2 Table. Annual emissions savings from avoided methane and increased carbon sequestration. This table summarizes values at each site calculated using Eq 1, where E are the annual emissions avoided, GWP is the global warming potential of methane, C is the increase in soil carbon sequestration, and VCUS-yr are the annual verified carbon units that could have been generated considering the CO2e potential of both avoided methane and increased carbon sequestered as a result of each restoration assuming that the project was considered for carbon credits via the Verified Carbon Standard. (DOCX) S3 Table. Social benefit [file NIHMS2015136-supplement-Supplement1.zip › pclm.0000317.s008.docx]

**S11 Table: Restored Marsh Salinity Variance by Year.** Of the six sites included in this analysis, shown here is the annual variance in salinity values demonstrated both before and after the first restoration year, which is demarcated by the grey highlighted cell for each site. Post-restoration sites that were below 18 ppt are demarcated by the orange highlighted cell and were not included in our estimates of avoided methane emissions since methane production during these years was possible. As is shown, average annual salinity values vary year-on-year both before and after implementation of the restoration project at each site. Further, this summation demonstrates a definitive lag phase in the Gloucester sites following the completed restoration project. Were this lag phase to have been included in our overall analysis, the total social benefit value for each site would have been reduced to only include years in which the average salinity was above 18 psu. Average pre- and post-salinity values vary here when compared against previous summary tables as these averages represent an *average of the annual averages* rather than an average of the pre- and post-restoration values.

| ***Year*** | ***Essex Conomo Point*** | ***Gloucester Eastern Point*** | ***Gloucester Mill Pond*** |  | ***Ipswich Cedar Rd.*** | ***Ipswich Town Farm*** | ***Rockport Saratoga*** |
| --- | --- | --- | --- | --- | --- | --- | --- |
| *1996* |  |  |  |  |  | *23.8 ± 1.04* |  |
| *1997* |  |  |  |  |  | *No Data* |  |
| *1998* | *20.7 ± 1.7* |  | *21 ± 0.8* |  |  | *29 ± 0.96* | *No Data* |
| *1999* | *15.7 ± 1.39* |  | *21.7 ± 1.33* |  | *12.6 ± 0.75* | *33.6 ± 2.66* | *No Data* |
| *2000* | *20 ± 1.72* | *12.1 ± 1.32* | *18.2 ± 0.71* |  | *10 ± 1.43* | *28 ± 1.28* | *No Data* |
| *2001* | *23.4 ± 0.96* | *13.2 ± 1.22* | *14.2 ± 0.68* |  | *5.1 ± 2.05* | *26 ± 1.3* | *No Data* |
| *2002* | *21.7 ± 1.46* | *10.9 ± 0.95* | *No Data* |  | *No Data* | *29.2 ± 0.96* | *18.2 ± 1.65* |
| *2003* | *25.5 ± 1.53* | *No Data* | *No Data* |  | *No Data* | *30.7 ± 1.12* | *No Data* |
| *2004* | *20.6 ± 0.67* | *16.3 ± 0.85* | *21 ± 2.96* |  | *7.3 ± 0.67* | *21.1 ± 1.12* | *13 ± 1.41* |
| *2005* | *23.2 ± 1.04* | *20.5 ± 1.11* | *16.2 ± 0.74* |  | *11 ± 4* | *24.3 ± 1.36* | *20.2 ± 1.66* |
| *2006* | *22.1 ± 1.18* | *18.6 ± 0.59* | *15 ± 0.63* |  | *8.5 ± 1.28* | *23.6 ± 1.01* | *11.2 ± 1.31* |
| *2007* | *24 ± 1.19* | *22.3 ± 1.66* | *18.4 ± 0.58* |  | *6.9 ± 0.68* | *24.5 ± 1.03* | *16.2 ± 1.29* |
| *2008* | *22.5 ± 1.21* | *19 ± 1.45* | *18.8 ± 0.91* |  | *6.5 ± 0.81* | *23.1 ± 1.16* | *10.2 ± 2.11* |
| *2009* | *19.3 ± 1.05* | *17.3 ± 1.15* | *19.3 ± 0.93* |  | *7.5 ± 1.27* | *23.3 ± 0.99* | *15.8 ± 1.75* |
| *2010* | *21.4 ± 0.73* | *19.1 ± 1.47* | *22.5 ± 1.21* |  | *14.9 ± 1.01* | *26.7 ± 0.96* | *22.3 ± 1.3* |
| *2011* | *19.4 ± 0.95* | *16.3 ± 1.59* | *20.7 ± 1.18* |  | *No Data* | *25.5 ± 1.2* | *12.9 ± 1.42* |
| *2012* | *24.6 ± 0.72* | *19.2 ± 1.26* | *16.5 ± 1.32* |  | *15.6 ± 1.32* | *28.4 ± 1.49* | *13.3 ± 0.98* |
| *2013* | *20.9 ± 0.8* | *20.4 ± 2.18* | *18.6 ± 1.04* |  | *10.9 ± 1.43* | *25.7 ± 0.95* | *17.7 ± 1.07* |
| *2014* | *25.8 ± 0.94* | *17.8 ± 1.77* | *22.6 ± 1.23* |  | *11.2 ± 4.23* | *24.5 ± 0.73* |  |
| *2015* | *29.1 ± 4.02* | *19.5 ± 1.84* | *25.1 ± 1.32* |  | *9.9 ± 1.44* | *25.9* |  |
| *2016* |  | *20 ± 1.28* | *29.9 ± 0.99* |  | *6.8 ± 1.23* | *29.8* |  |
| *2017* |  | *20.8 ± 1* | *25.1 ± 0.87* |  | *10.9 ± 1.45* | *27.3* |  |
| *2018* |  | *18.5 ± 0.63* | *21 ± 1.12* |  | *15.1 ± 1.53* | *28.2* |  |
| *Avg. Pre* | *18.2 ± 1.54* | *12.1 ± 1.16* | *18.8 ± 0.88* |  | *12.6 ± 0.75* | *27.7 ± 1.31* | *18.2 ± 1.65* |
| *Avg. Post* | *22.9 ± 1.25* | *19.2 ± 1.36* | *20.7 ± 1.14* |  | *9.9 ± 1.63* | *25.9 ± 1.06* | *15.3 ± 1.43* |
